# Pyrenol in the Treatment of Influenza

**Published:** 1907-05

**Authors:** 


### ABSTRACTS.

### Pyrenol in the Treatment of Influenza

IN the Berliner klin, Wochenschrift, Feb. 11, 1907, Braeunig refers to the paper by Steiner on the pyrenol treatment of influenza (*Fortschritte d. Medizin*, Vol. 23, No. 15). Steiner found in hospital and private practice that pyrenol reduces the temperature and leads to a rapid termination of the process free from complications. Similarly, it acts as an antipyretic and sedative in pertussis, also influencing the diseased mucosa, and thus fulfils two indications at once. He especially emphasises the absolute harmlessness of the remedy, even with children of less than one year.

Braeunig adds that the paper tells nothing new, for the beneficial effect of pyrenol in these diseases is well known. He also has found it to do good service in similar cases, as well as in the hectic fever of phthisis in which,—given at the right time,—it forestalls the high evening temperatures, without causing the very annoying diaphoresis which follows the use of other antipyretics.

